# First person – Tiyasha Sarkar

**DOI:** 10.1242/bio.056267

**Published:** 2020-09-24

## Abstract

First Person is a series of interviews with the first authors of a selection of papers published in Biology Open, helping early-career researchers promote themselves alongside their papers. Tiyasha Sarkar is first author on ‘Neuronal changes and cognitive deficits in a multi-hit rat model following cumulative impact of early life stressors’, published in BiO. Tiyasha is a PhD scholar in the lab of Professor Ishan Kumar Patro at the School of Studies in Neuroscience, Jiwaji University Gwalior, Madhya Pradesh, India, investigating the correlation between multiple early life stressors and later life neurological disorders.


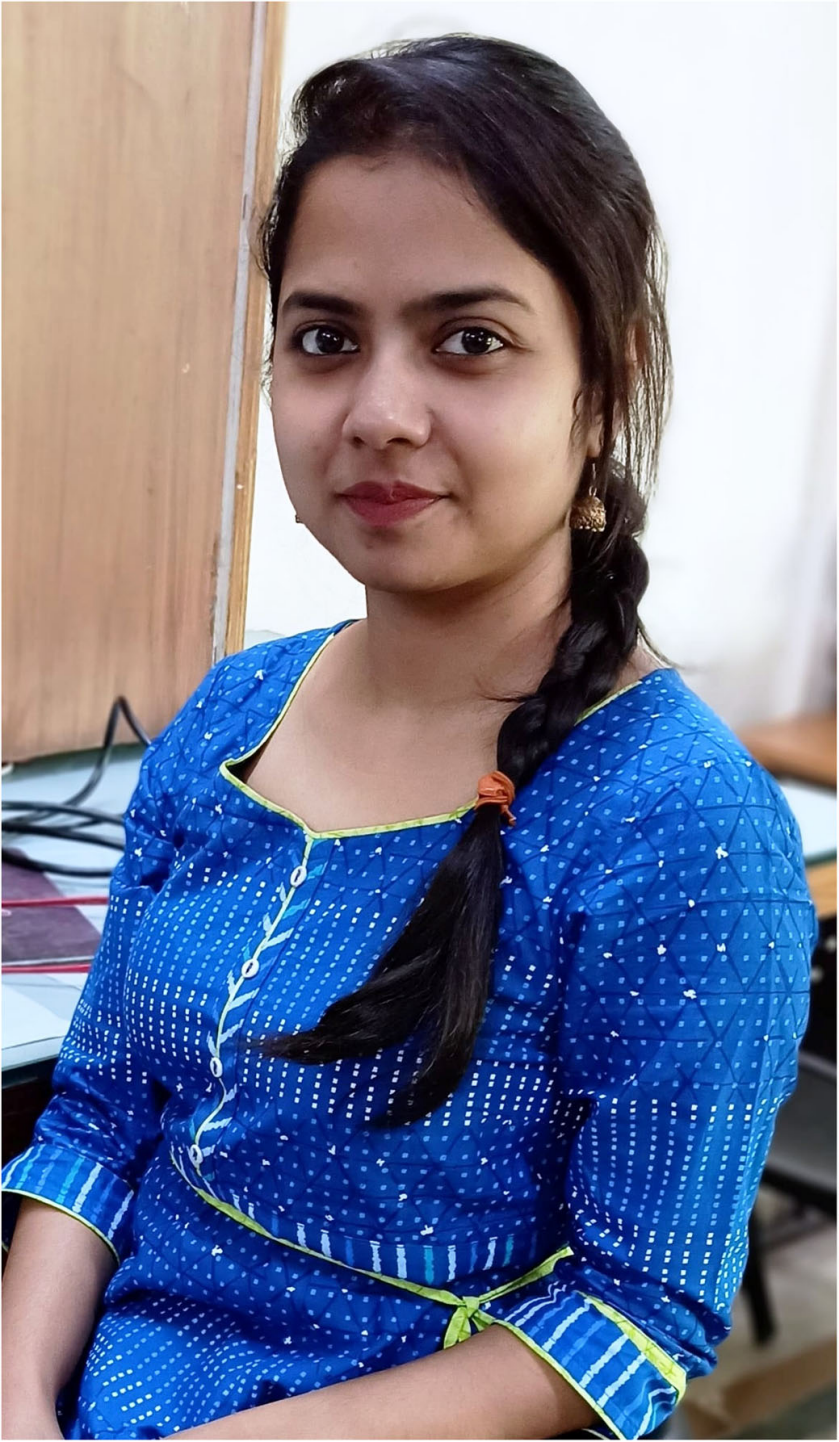


**Tiyasha Sarkar**

**What is your scientific background and the general focus of your lab?**

Our lab deals with the effect of major early-life stressors like toxins, protein malnourishment, and viral and bacterial infections on the nervous system. The long-term consequences of such stressors on the brain as well as cognitive and behavioural abilities of the effected individuals are being studied with respect to cellular changes in the brain. Both singular and combined exposures of stressors (multi-hit) are used to study cellular, cognitive and behavioural changes in rat models.

**How would you explain the main findings of your paper to non-scientific family and friends?**

During the early years of an individual's life they are prone to various environmental insults like malnourishment, infections and toxins. Maternal protein malnourishment occurs in a majority of expecting mothers from poor socioeconomic countries, and children born to such mothers are immune compromised. Poor immunity makes the children susceptible to further viral and bacterial infections. All such stressors act synergistically and change the cellular architecture of the brain, leading to learning and memory deficiency in adult individuals, which might be the reason for the development of various later life neurological disorders.

**What are the potential implications of these results for your field of research?**

The majority of studies that are available only talk about a single type of stress and their effect on the nervous system. This is a vertical study that, for the very first time, reported the consequences of multiple stressors encountered simultaneously by an individual at an early age. This data will further help researchers to consider the overall scenario of conditions suffered by people belonging to low socioeconomic backgrounds, in which people suffer from more than one type of stressor simultaneously. This could help to find the proper link between early life stressors and later life neurological disorders.

**What has surprised you the most while conducting your research?**

The most surprising thing we noticed was the consequences of multi-hit. While everyone was talking about single stress, we found that multi-hit accelerated the cellular damage in brain many fold and could be the actual reason for the development of neurological disorders. Environmental insults work together in a cascade and change the cytoarchitecture of the brain, which further damages cognitive and behavioural abilities, the common phenotype of almost every neurological disorder.

“Environmental insults work together in a cascade and change the cytoarchitecture of the brain…”

**What, in your opinion, are some of the greatest achievements in your field and how has this influenced your research?**

The greatest achievements in our field will be the discovery of the cellular structures and the mechanisms how cells crosstalk and communicate. The more we see the more we want to explore and hence, the details of brain cells that are available helped us to understand the mechanism and how is it altered on any type of environmental insults.

**Figure:**
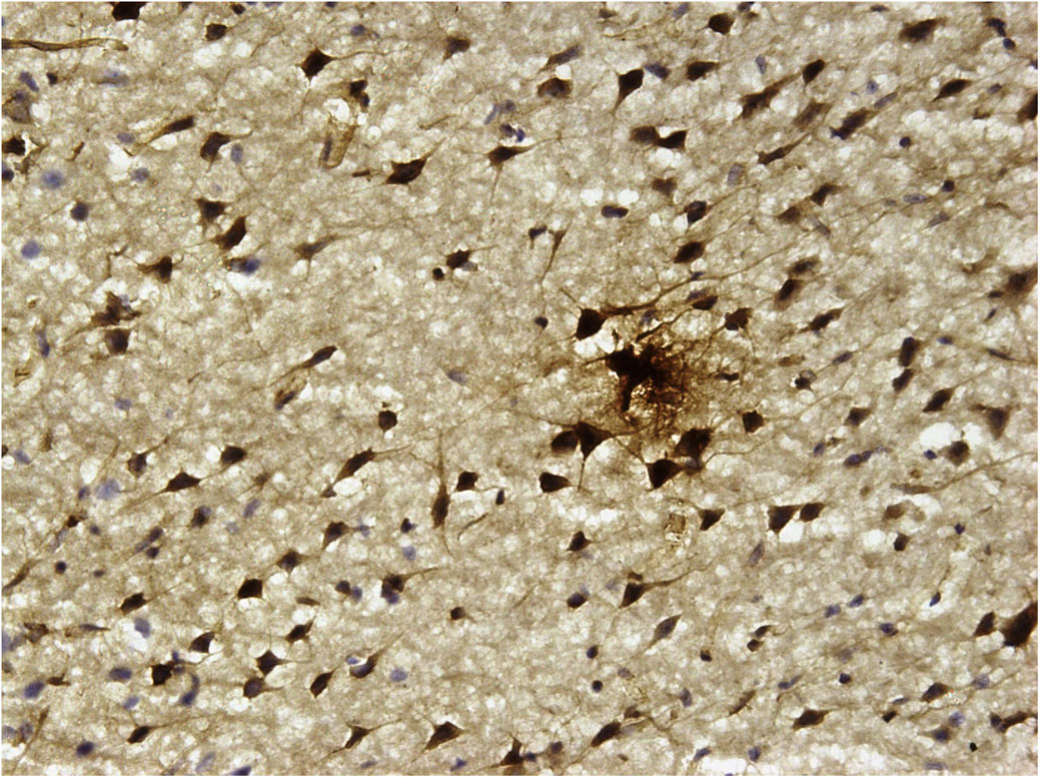
**Doublecortin (DCX) positive naive neurons in the cortex of adult rats treated with multiple early life stressors.**

**What changes do you think could improve the professional lives of early-career scientists?**

Lowering stress levels and negative competition could definitely improve the professional lives of early-career scientists.

**What's next for you?**

After finishing my PhD, I want continue my research in the field of neuroscience and would like to work as a postdoc fellow.
